# Prediction intervals reveal uncertainty in the effect of metabolic syndrome on surgical mortality: a closer look at heterogeneity

**DOI:** 10.1097/JS9.0000000000002004

**Published:** 2024-08-02

**Authors:** I-Wen Chen, Wei-Ting Wang, Chien-Ming Lin, Kuo-Chuan Hung

**Affiliations:** a Department of Anesthesiology, Chi Mei Medical Center, Liouying, Tainan city; b Department of Anesthesiology, E-Da Hospital, I-Shou University, Kaohsiung City; c Department of Anesthesiology, Chi Mei Medical Center, Tainan city, Taiwan

*Dear Editor,*

We read with great interest the article “Metabolic syndrome and surgical complications: a systematic review and meta-analysis of 13 million individuals” recently published in the International Journal of Surgery by Norris *et al.* ^1^. The authors performed an expansive systematic review and meta-analysis to evaluate the association between metabolic syndrome (MetS) and 30-day postoperative complications. Through a rigorous search, 63 studies encompassing over 13 million patients who met the eligibility criteria were identified. The meta-analysis found that compared to individuals without MetS, those with MetS had a 75% increased risk of 30-day mortality, 64% higher risk of surgical site infections, 56% greater risk of cardiovascular events, and 55% increased odds of hospital readmission after surgery^1^. We want to congratulate the authors on conducting such a large-scale, well-designed review and synthesizing current evidence related to surgical outcomes in patients with metabolic disease. The inclusion of over 13 million participants, spanning nearly all surgical specialties and geographic regions, substantially strengthened the reliability and generalizability of the results.

Despite the inclusion of such a large number of patients, the significant heterogeneity (i.e. I^2^=93%) observed in the primary outcome (i.e. the risk of 30-day mortality)^1^ raises concerns. Although the causes of heterogeneity were not specifically analyzed, differing study populations, designs, confounders, and surgical procedures were likely contributing factors. For example, differences in patient characteristics, such as age, sex, ethnicity, and comorbidities, between these populations could contribute to heterogeneity in the results. In addition, this meta-analysis included both retrospective and prospective observational studies. Retrospective studies are more prone to selection bias and confounding factors than prospective studies. Furthermore, a wide range of surgical procedures were included, including orthopedic, cardiac, vascular, and bariatric procedures. The relationship between metabolic syndrome and surgical outcomes may differ depending on the type of surgery and may be a major source of heterogeneity. The authors^1^ used random-effects models to account for statistical heterogeneity, but this variability might hinder the interpretation of the findings. Given this heterogeneity, further analyses, including sensitivity or subgroup analyses, should be performed to better explore the source of heterogeneity.

Additionally, the analysis of prediction intervals can be used to estimate the range of true effects that will likely be observed in similar future studies^2,3^. Unlike confidence intervals, which estimate the precision of the summary effect, prediction intervals reflect heterogeneity^2,3^. When a prediction interval crosses 1, it indicates that the direction of the effect may flip in similar future studies. In some cases, exposure (e.g. MetS) could be protective, whereas in others, it is a risk factor for the outcome (e.g. mortality). Prediction intervals caution us against overinterpretation and provide valuable insights into the expected variation in results across different settings. This will provide readers with a better understanding of the certainty and clinical applicability of the association between MetS and surgical complications. To address the issue of high heterogeneity, we analyzed prediction intervals based on raw data from the original meta-analysis^1^. Comprehensive Meta-Analysis (Version 4, Biostat) was used for the analysis, as previously reported^4,5^. Figure 1 shows a significant variation in the prediction interval for 30-day mortality outcomes, ranging from 0.44 to 6.99. This implies that in similar future studies, the real effect could substantially differ, potentially showing either beneficial or harmful outcomes.

**Figure 1:**
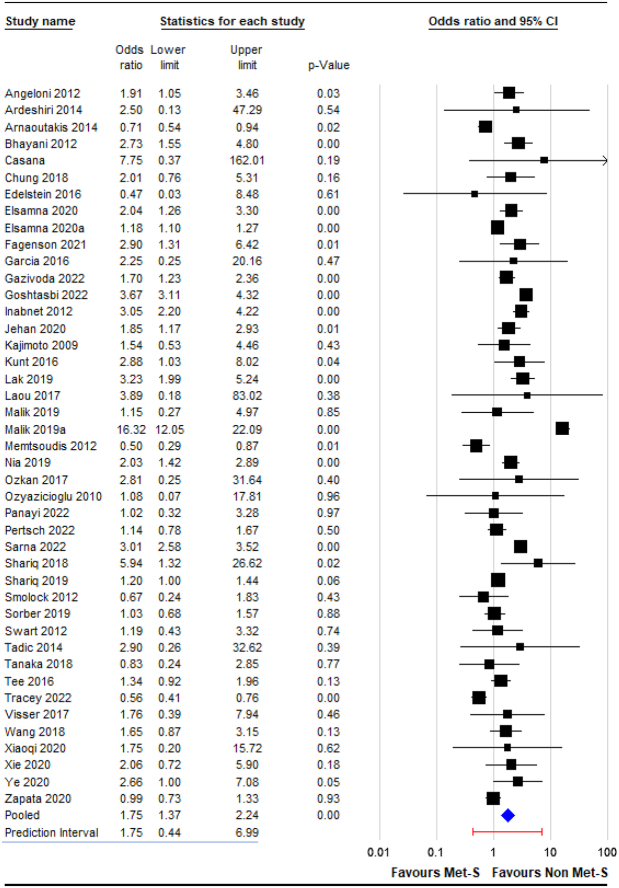
Prediction interval for the effect of metabolic syndrome (MetS) on postoperative mortality risk. The blue curve represents the distribution of true effects, with the peak indicating the most likely effect size (odds ratio of 1.75), and the tails showing the range of possible effects that could be observed in 95% of similar future studies. The prediction interval spans from 0.44 to 6.99, suggesting that while MetS increases mortality risk on average, in some settings, it may be protective (odds ratio < 1), whereas in others, it could substantially increase risk (odds ratio > 7). This wide interval crossing the null value of 1 indicated uncertainty in the consistency of the effect across all populations and limited definitive conclusions regarding the impact of MetS on surgical mortality risk.

In conclusion, given this uncertainty, it would be premature to directly apply these results to guide clinical practice without further confirmation. Nevertheless, the findings of the original meta-analysis^1^ suggest a potentially higher risk of complications in patients with MetS, warranting clinicians’ attention. Additional rigorous analyses accounting for heterogeneity and consolidating evidence across multiple settings are warranted before making definitive practice recommendations for surgical populations with MetS.

## Ethical approval

Not applicable.

## Consent

Not applicable.

## Source of funding

No external funding was received for this study.

## Author contribution

I.-W.C. and K.-C.H. wrote the main manuscript text. W.-T.W. and C.-M.L. prepared Figure 1. All authors read and approved the final version of the manuscript.

## Conflicts of interest disclosure

The authors declare no conflicts of interest.

## Research registration unique identifying number (UIN)

Not applicable.

## Guarantor

Kuo-Chuan Hung.

## Data availability statement

The datasets used and/or analyzed in the current study are available from the corresponding author upon reasonable request.

## Provenance and peer review

This paper was not invited.
